# Case Report: A case report of acromegaly associated with primary aldosteronism

**DOI:** 10.12688/f1000research.3-58.v2

**Published:** 2014-06-18

**Authors:** Joanna Matrozova, Silvia Vandeva, Sabina Zacharieva

**Affiliations:** 1 Clinical Center of Endocrinology and Gerontology, Medical University – Sofia, 1431, Sofia, Bulgaria

## Abstract

We describe a patient with a rare combination of acromegaly and primary aldosteronism. A 37 year-old female patient was diagnosed with acromegaly on the basis of typical clinical, hormonal and image characteristics. She presented also with one of the most common co-morbidities – arterial hypertension. The patient has been regularly followed-up and after three surgical interventions, irradiation and adjuvant treatment with a dopamine agonist, acromegaly was finally controlled in 2008 (20 years after diagnosis). Arterial hypertension however, remained a therapeutic problem even after prescription of four antihypertensive drugs. She had normal biochemical parameters, except for low potassium levels 3.2 (3.5-5.6) mmol/l. This raised the suspicion of primary hyperaldosteronism, confirmed by a high aldosterone to plasma rennin activity ratio, high aldosterone level after a Captopril challenge test and visualization of a 35 mm left adrenal nodule on a CT scan. After an operation, the patient recovered from hypokalemia and antihypertensive therapy was reduced to a small dose of a Ca blocker.

Co-morbid arterial hypertension is common in acromegaly, though it is rare for this to be caused by Conn’s adenoma. The association of Conn’s adenoma with acromegaly has been interpreted in two lines: as a component of multiple endocrine neoplasia type (MEN1) syndrome or as a direct mitogenic effect of hyperactivated GH-IGF1 axis.

## Introduction

In the literature several patients with primary aldosteronism (PA) associated with endocrine and non-endocrine tumors have been reported ^1– 3^. These cases were attributed mainly to multiple endocrine neoplasia type 1 (MEN1), which is an autosomal dominantly inherited condition, characterized by the association of tumors of the pituitary and the parathyroid glands, the endocrine pancreas, the adrenal glands and neuroendocrine tumors ^4^. Aldosterone-producing adenomas have been described in various combinations, mainly with hyperparathyroidism, prolactinomas and pancreatic endocrine tumors, but none of these have been associated with acromegaly ^2, 3, 5^.

On the other hand, the prevalence of adrenal lesions in sporadic cases of acromegaly is higher than in the general population, possibly due to the permissive role of growth hormone (GH) and insulin-like growth factor 1 (IGH1) on tumorigenesis ^6– 8^. Previously benign, non-secreting adrenal tumors have been described in patients with acromegaly ^6– 8^. However, to our knowledge the association between acromegaly and Conn’s adenoma in sporadic acromegaly has been reported only in isolated cases ^9, 10^.

We describe herein a case of acromegaly, associated with PA due to an aldosterone-producing adenoma of the adrenal gland.

## Case report

In May 2008, a 57 year-old, Caucasian woman with a 20 year history of acromegaly was hospitalized in our clinic for a regular follow-up. Acromegaly was diagnosed in 1988 on the basis of a GH of 9.3 mIU/L, which was not suppressed during a glucose tolerance test (OGTT; GH 13.6 mIU/L) and a macroadenoma of the pituitary gland visualized on CT. Prolactin levels were also high: 3300 mIU/l (normal value <650). A non-radical transsphenoidal adenomectomy was performed in the same year and another one in 1991 after growth of the remnant tumor mass. Histology conducted in 1988 showed somatoprolactinoma. In 1993, she had a transcranial adenomectomy due to remnant macroadenoma. As the disease activity (i.e. GH hypersecretion and presence of tumor mass) still persisted, in 1994 she was treated with radiotherapy and in the period between 1994 and 2001 she received dopamine agonists (15 mg daily of bromocriptine for six years and 2×0.5 mg weekly cabergoline for the last four months of the period). Acromegaly was still not controlled and a treatment with a somatostatin analogue (vapreotide s.c. implant, 396 mg every 12 weeks) was started in 2001. The patient was estimated to be a partial responder with no significant improvement in hormonal parameters, so she was switched back to 2×0.5 mg weekly dose of cabergoline. The MRI from 2008 showed a tumor remnant of 18 mm, spreading towards the right part of the sphenoidal sinus.

The patient had a past medical history of multinodular goiter, operated on in 2001 and recurrent in 2008. She has been hypertensive since she was 37 years old. Her hypertension has never been well controlled on a triple therapy, including a diuretic (enalapril maleate 2×20 mg, nifedipine 4×10 mg and chlortalidone 100 mg daily). She was operated on for a colon polyp in 1994 and was followed-up for myoma of the uterus. Family history of hypertension was recorded with both parents being hypertensive.

On admission in 2008 she was 1.70 m tall, weighed 83 kg with a waist circumference of 89 cm and had a BMI of 29. Her blood pressure was 150/90 mmHg. Arterial blood pressure monitoring (ABPM) showed a mean daily systolic blood pressure (SBP) of 150 mmHg and diastolic blood pressure (DBP) of 93 mmHg. At that time she was treated with nitrendipine 20 mg twice a day, enalapril 20 mg twice a day, indapamide 2.5 mg per day, prazosin 0.5 mg per day, and cabergoline 2×0.5 mg weekly. Physical examination showed typical acromegalic features such as enlargement of the hands and feet, deep nasolabial folds and macroglossia.

**Figure 1.**
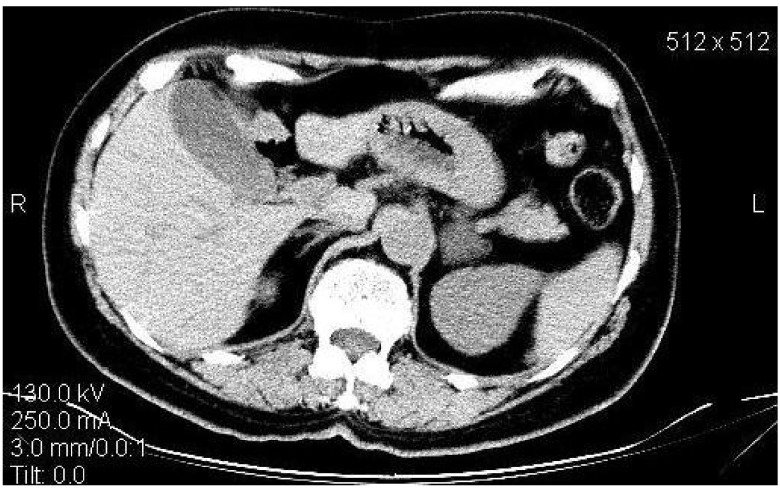
CT scan visualizing 35 mm tumor in the left adrenal gland and a normal right adrenal gland.

The association of resistant hypertension and hypokalemia raised the suspicion of PA. Baseline laboratory data are shown in Table 1. Treatment with ACE-inhibitors and diuretics was stopped for 20 days to investigate her Renin-Angiotensin-Aldosterone-System (RAAS) before the next presentation to our clinic. In our unit the aldosterone to renin ratio (ARR) is used as a screening test for PA. In patients with elevated basal ARR (>750 pmol/l per ng/ml/h) a confirmatory test (Captopril challenge test) is performed. The diagnosis of PA is confirmed if the aldosterone is >330 pmol/l at the 90th minute after the oral administration of 50 mg of Captopril, with the patient asked to remain in a sitting position throughout the test. In our patient the basal and post-captopril aldosterone to renin ratio was 1260 and 3545, respectively. Suppressed plasma rennin activity (PRA) was measured at the beginning and the end of the test (<0.2 ng/ml, normal range 0.3–3). Basal aldosterone was 252 pmol/l and 709 pmol/l at the end of the test. The adrenal CT scan showed a 35 mm nodule in the left adrenal gland ( Figure 1) and aldosterone-producing adenoma (Conn’s adenoma) was diagnosed. The patient was operated on in August 2008. A tumor of 35 mm was found and histology (hematoxylin and eosin) data showed a tumor of adrenal cortex origin, consisting of light cells.

**Table 1.** Biochemical and hormonal parameters at diagnosis of primary aldosteronism.

Parameter	Result	Normal range
Fasting blood glucose, mmol/l	4.62	3.89–6.1
Total cholesterol, mmol/l	5.35	<5.2
Triglycerides, mmol/l	0.78	<1.72
HDL cholesterol, mmol/l	1.68	>1.3
Creatinine, µmol/l	53	<106
Potassium, mmol/l	3.2–3.6	3.5–5.6
IGF1, nmol/l	26.8	14–40.5
GH in the course of glucose tolerance test, mUI/l	0 min 3.7 60 min 2.9 120 min 2.1	GH<5 mIU/l (2 ng/ml) at baseline and GH<2.5 mIU/l (1 ng/ml) during OGTT
FT4, pmol/l	12.5	9–23

IGF1- insulin-like growth factor-1, GH- growth hormone, FT4- free thyroxine

The patient had a follow-up visit three months after the operation. Recovery from hypokalemia was recorded (potassium: 4.95 mmol/l). Over this period the antihypertensive therapy was reduced and she was receiving only a small dose of a Ca blocker (nitrendipine 40 mg daily). Her blood pressure was 130/80 mmHg. ABPM showed a mean daily SBP of 138 mmHg and a DBP of 83 mmHg, and a mean heart rate of 53 bpm. A Captopril test was performed which revealed normal aldosterone levels with a tendency to low renin values: 0 min PRA 0.3 ng/ml/h, aldosterone 82 pmol/l, ARR 273; 90 min PRA 0.3 ng/ml/h, aldosterone 98.9 pmol/l, ARR 329.

In the last three years of her regular follow-up visits the patient was found to be normokalemic and normotensive on a double antihypertensive therapy (ACE-inhibitor and Ca blocker). Her most recent visit to our unit was in June 2013. Acromegaly was well controlled on cabergoline 2×0.5 mg weekly. Hormonal parameters showed IGF-1 to be 20.1 nmol/l (normal range: 14–40.5), serum GH during OGTT was: 0 min 2.7 mUI/l; 60 min 1.6 mUI/l; 120 min 1.4 mUI/l (criteria for acromegaly control: basal GH<1 ng/ml (3 mIU/l) and <0.4 ng/ml (1.2 mIU/l) during OGTT ^11^). The last MRI of the pituitary gland, performed in 2010, did not show any enlargement of the existing tumor remnant. During the most recent visit in June 2013 the patient had slightly elevated blood pressure, well controlled using small doses of an ACE- inhibitor (10 mg daily lisinopril) and a Ca-blocker (20 mg daily nifedipine). Laboratory data showed normal potassium of 5.1 mmol/l. The association of acromegaly and Conn’s adenoma raised the hypothesis for MEN1 in our patient, therefore the parathyroid glands were investigated using ultrasound. There were no clinical, laboratory or ultrasound data for primary hyperparathyroidism. Biochemical parameters were normal: serum calcium levels were 2.42 mmol/l (normal range: 2.12–2.62) and phosphorus was 1.35 (normal range: 0.87–1.45). The ultrasound image was not suspicious for parathyroid adenoma. The patient had no relatives with endocrine tumors.

## Discussion

We describe a rare combination of acromegaly and PA due to an aldosterone-producing adenoma. There are several recent reports which describe the association of PA and different types of tumors ^1– 3^. These cases are related mainly to MEN1. MEN1 is caused by germline mutations of the *menin* gene, which most frequently leads to the development of primary hyperparathyroidism, pituitary adenomas and pancreatic tumors. The prevalence of pituitary tumors in MEN1 varies according to different studies, from 10 to 76% ^12– 14^. Somatotropinomas causing acromegaly occur in 3–6% of MEN1 patients ^14, 15^. As for adrenal involvement in patients with MEN1, the data are contradictory with a prevalence ranging from 9 to 73% depending on the investigated series ^16– 18^. A recent large study in 715 MEN1 patients showed adrenal lesions in 146 cases, and among them 72 had adrenal adenomas, comprising 10% of the whole cohort ^5^. Four cases of PA were found in the whole group. None of them was associated with acromegaly, but rather with primary hyperparathyroidism, prolactinoma, or pancreatic endocrine tumors.

In the literature only single cases of PA have been described in MEN1 ^1– 3^. Beckers *et al.* ^1^ reported a case of PA as a part of MEN1, associated with parathyroid adenoma, prolactinoma and toxic multinodular goiter. Kim *et al.* ^2^ described a case of PA, associated with primary hyperparathyroidism, Hurthle cell thyroid cancer and meningioma with a loss of heterozygosity (LOH) of the *MEN1* locus in parathyroid glands, but no germline mutation. Honda *et al.* ^3^ reported PA associated with primary hyperparathyroidism and breast cancer and a LOH of the MEN1 locus in the parathyroid adenoma and in the breast cancer tissue. None of the reported cases was associated with acromegaly, in contrast to our patient. Although investigations for primary hyperparathyroidism were negative in our case, the patient showed two endocrine tumors over several years, which could be related to the presence of MEN1. This could not be confirmed as genetic analysis was not performed due to funding restrictions, which is a serious weak point of the case presentation. However, in different series in up to 60% of patients with a sporadic MEN1 phenotype, no mutation of the corresponding *menin* gene has been found ^19^.

On the other hand we could speculate that the activated GH-IGF1 axis may have a role in the morphological and functional adrenal changes in aldosterone-producing adenomas in acromegaly. Although the incidence of neoplasms in acromegaly is a matter of debate, numerous reports have suggested that patients are at increased risk of developing thyroid nodules and colon polyps ^20^, as well as thyroid cancer ^21^ breast cancer ^22^ and colorectal cancer ^23^. There are limited data in the literature concerning the adrenal involvement in acromegalic patients. In a study by Scaroni *et al.* adrenal morphological abnormalities were found in 28.7% of acromegalic patients (n=94), among them nine cases with unilateral adenoma and the rest with uni- or bilateral hyperplasia ^6^. All tumors were hormonally inactive and no cases of PA were found.

Another recent study found an even higher prevalence of adrenal abnormalities in patients with acromegaly, describing abnormal adrenal morphology in 48% of patients in a group with 670 acromegalics. Among them 19 patients had an adrenal adenoma, 10 subjects had adrenal hyperplasia and 7% had hyperaldosteronism ^7^. The increased prevalence of adrenal incidentaloma in acromegaly suggests that GH and IGF1 may have an effect on adrenal morphological changes, although in both studies cited above, no significant correlation with GH-IGF1 were found. On the other hand, it has been shown that IGF1 factors are potent mitogens and a strong IGFII expression has been demonstrated in adrenocortical tumors ^24^. Also, a recent study demonstrated that the GH receptor is expressed in both normal rat and normal and diseased human adrenals, which suggests direct action of GH in adrenal tissue ^25^.

From a functional point of view several studies have explored the interactions between the GH-IGF1 axis and RAAS. A stimulatory effect of the GH/IGF1 on RAAS has been demonstrated in some reports ^8, 9, 26^. A recent study by Bielohuby *et al.* ^27^ showed increased levels of aldosterone in acromegalic patients which normalized after surgery. Aldosterone levels were elevated in a transgenic mouse model over-expressing GH compared to non-transgenic mice and changes in aldosterone were independent of IGF1, renin and the expression of aldosterone synthase ^27^. These studies suggest a direct effect of GH on adrenal glands, which could lead to abnormalities of function or morphology and eventually to the formation of an aldosterone-producing adenoma as may have occurred in our patient. Finally, we could not exclude the possibility of a mere coincidence in the co-existance of both pathologies.

## Conclusion

We have described an uncommon case of two endocrine tumors – somatoprolactinoma and Conn’s adenoma diagnosed years after initial presentation of acromegaly. This combination could be part of the MEN1 syndrome, despite the absence of hyperparathyroidism. On the other hand, our patient already had several co-morbidities due to the mitogenic effect of the GH-IGF1 hyperactivation (multinodular goiter, myoma and colon polyp), which is another plausible hypothesis for the functional and structural changes in the adrenal gland.

## Consent

Written informed consent for publication of clinical details and clinical images was obtained from the patient.
