# Supplementary material for: Case Report: A case report of acromegaly associated with primary aldosteronism
Source: F1000Res. 2014 Jun 18;3:58. Originally published 2014 Feb 14. [Version 2] doi: 10.12688/f1000research.3-58.v2 (PMC4156025; doi:10.12688/f1000research.3-58.v2)
Supplement: Peer review history

# Peer review: Referee response for version 2

**DOI:** 10.5256/f1000research.4622.r5242

**Published:** 2014-06-26

**Authors:** Niki Karavitaki

**Affiliations:** 1 Department of Endocrinology, Oxford Centre for Diabetes, The Churchill Hospital, University of Oxford, Oxford, UK

The presented case is interesting.

I have read this submission. I believe that I have an appropriate level of expertise to confirm that it is of an acceptable scientific standard.

# Peer review: Referee response for version 2

**DOI:** 10.5256/f1000research.4622.r5243

**Published:** 2014-06-25

**Authors:** Shlomo Melmed

**Affiliations:** 1 Pituitary Center, Department of Medicine, Cedars-Sinai Medical Center, Los Angeles, CA, USA

I have read this submission. I believe that I have an appropriate level of expertise to confirm that it is of an acceptable scientific standard.

# Peer review: Referee response for version 1

**DOI:** 10.5256/f1000research.3454.r4554

**Published:** 2014-04-25

**Authors:** Shlomo Melmed

**Affiliations:** 1 Pituitary Center, Department of Medicine, Cedars-Sinai Medical Center, Los Angeles, CA, USA

This is a well described case of interest to readers. It is clearly written and contains important information.

I have read this submission. I believe that I have an appropriate level of expertise to confirm that it is of an acceptable scientific standard.

# Response

**Published:** 2014-05-22

**Authors:** Silvia Vandeva

**Affiliations:** Clinical Center of Endocrinology and Gerontology 'Akad Iv Pentchev', Sofia Medical University, Bulgaria

Dear prof. Melmed,

The authors thank you for your comments.

# Peer review: Referee response for version 1

**DOI:** 10.5256/f1000research.3454.r3909

**Published:** 2014-03-10

**Authors:** Niki Karavitaki

**Affiliations:** 1 Department of Endocrinology, Oxford Centre for Diabetes, The Churchill Hospital, University of Oxford, Oxford, UK

This is an interesting case of a patient with co-existing acromegaly and primary aldosteronism attributed to a Conn's adenoma.

Although the biochemical confirmation of primary aldosteronism relied only on a Captopril test and no adrenal vein sampling took place, the improvement of the blood pressure control and the resolution of hypokalaemia following the removal of the adrenal mass support the diagnosis of Conn's adenoma.

The authors attempt to provide a pathophysiological link between the two conditions. Their thoughts are very interesting but the option of a mere coincidence also needs to be mentioned.

I have read this submission. I believe that I have an appropriate level of expertise to confirm that it is of an acceptable scientific standard.

# Response

**Published:** 2014-05-22

**Authors:** Silvia Vandeva

**Affiliations:** Clinical Center of Endocrinology and Gerontology 'Akad Iv Pentchev', Sofia Medical University, Bulgaria

Dear Dr Karavitaki,

The authors thank you for your comments. We have conformed to your recommendations.
